# Supplementary material for: Current Physical Therapy for Skin Scar Management: A Scoping Review
Source: J Clin Med. 2025 Aug 22;14(17):5920. doi: 10.3390/jcm14175920 (PMC12429749; doi:10.3390/jcm14175920)
Supplement: Supplementary file 1 [file jcm-14-05920-s001.zip › Supplementary Material S1.pdf]

Current Physical Therapy for Skin Scar Management: A Scoping Review

American Journal of Clinical Dermatology

Sara Di Serio, Matteo Congiu[*], Silvia Minnucci, Valentina Scalise, Firas Mourad

*Correspondence: Matteo Congiu, Department of Neuromotor Rehabilitation, IRCCS Istituto Auxologico Italiano, Milan, Italy. Email: matteo.congiu@students.uniroma2.eu

**Embase**
'Physiotherapy' OR 'Rehabilitation' OR 'Manipulative Medicine' OR 'Resistance Training' OR 'Patient Education' OR 'Mobilization' OR 'Conservative Treatment' OR 'Non Invasive Procedure' OR 'Soft Tissue Therapy' OR 'Exercise'
'Scar'/Exp OR 'Scar' OR 'Hypertrophic Scar'/Exp OR 'Hypertrophic Scar' OR 'Keloid'/Exp OR 'Keloid' OR 'Tissue Adhesion'/Exp OR 'Tissue Adhesion'

**Cochrane**
#1 MeSH descriptor: [Cicatrix] explode all trees<br>#2 MeSH descriptor: [Cicatrix, Hypertrophic] explode all trees<br>#3 MeSH descriptor: [Tissue Adhesives] explode all trees<br>#4 MeSH descriptor: [Keloid] explode all trees<br>#5 "Burn scar" OR "Scar" OR "Contracture Scar" OR "Cicatrix" OR "keloid"<br>#6 #1 OR #2 OR #3 OR #4 OR #5 8676<br>#7 MeSH descriptor: [Rehabilitation] explode all trees<br>#8 MeSH descriptor: [Physical Therapy Modalities] explode all trees<br>#9 MeSH descriptor: [Exercise] explode all trees<br>#10 MeSH descriptor: [Patient Education as Topic] explode all trees<br>#11 MeSH descriptor: [Musculoskeletal Manipulations] explode all trees<br>#12 MeSH descriptor: [Therapy, Soft Tissue] explode all trees<br>#13 MeSH descriptor: [Self Care] explode all trees<br>#14 "Physiotherapy" OR "Physical Therapy" OR "Exercise" OR "Rehabilitation" OR "Manual Therapy" OR "Patient Education"<br>#15 #7 OR #8 OR #9 OR #10 OR #11 OR #12 OR #13 OR #14<br>#16 #6 AND #15

**CINAHL**
(MH "Cicatrix, Hypertrophic") OR (MH "Cicatrix+") OR (MH "Keloid") OR ("Scar*") OR ("Burn Scar*") OR ("Scarring") OR ("Tissue adhesions") OR ("Hypertrophic scar*") OR ("Cicatrix")
("Stretching") OR ("Resistance Training") OR ("Muscle Strengthening") OR ("Mobilization") OR ("Conservative Treatment") OR ("Noninvasive Treatment") OR ("Exercise") OR ("Therapeutic Exercise") OR ("Patient Education") OR ("Rehabilitation") OR ("Physical Therapy") OR ("Physiotherapy") OR ("Manual Therapy") OR ("Self Care") OR (MM "Stretching") OR (MM "Resistance Training") OR (MM "Muscle Strengthening") OR (MM "Joint Mobilization") OR (MM "Mobility Training") OR (MM "Conservative Treatment") OR (MM "Noninvasive Procedures") OR (MM "Physical Therapy") OR (MM "Home Physical Therapy") OR (MM "Education, Physical Therapy") OR (MM "Physical Therapy Practice, Research-Based") OR (MM "Manual Therapy") OR

(MM "Patient Education") OR (MM "Patient Discharge Education") OR (MM "Self-Management") OR (MM "Self Care") OR (MM "Exercise") OR (MM "Therapeutic Exercise") OR (MM "Rehabilitation") OR (MM "Home Rehabilitation") OR (MM "Rehabilitation Patients")

**PEDro**
Abstract & Title:<br>- Physical therapy<br>- Physiotherapy<br>- Rehabilitation<br>- Education<br>- Self care<br>- Exercise<br>- Resistance training<br>- Strengthening<br>- Manual therapy<br>- Mobilization<br>- Soft tissue<br>- Stretching<br>- Conservative treatment<br>- Noninvasive treatment<br>- Scar
Therapy:<br>- Stretching, mobilisation, manipulation, massage<br>- Strength training<br>- Education
Subdiscipline:<br>- Musculoskeletal<br>- Orthopaedics

**PubMed**
“Cicatrix"[Mesh], "Cicatrix, Hypertrophic"[Mesh], "Tissue Adhesions"[Mesh], "Keloid"[Mesh], “Scar”, “Scarring”, “Hypertrophic Scars”, “Burn Scar”, “Contracture Scar”
"Rehabilitation"[Mesh], "Exercise Therapy"[Mesh], "Physical Therapy Modalities"[Mesh], "Musculoskeletal Manipulations"[Mesh], "Therapy, Soft Tissue"[Mesh], "Exercise"[Mesh], "Self Care"[Mesh], “Patient education as topic[Mesh], "Muscle Stretching Exercises"[Mesh], “Habilitation”, “Physiotherapy”, “Physical Therapy”, “Manual Therapy”, “Patient Education”, “Mobilization”

**Google Scholar**
(“Scar” OR “Scarring” OR “Hypertrophic Scars” OR “Burn Scar” OR “Contracture Scar” OR "Keloid") AND ("Rehabilitation" OR "Exercise Therapy" OR "Physical Therapy Modalities" OR "Musculoskeletal Manipulations" OR "Therapy, Soft Tissue" OR "Exercise" OR "Self Care" OR “Patient education as topic" OR "Muscle Stretching Exercises" OR “Habilitation” OR

“Physiotherapy” OR “Physical Therapy” OR “Manual Therapy” OR “Patient Education” OR “Mobilization”)
